# A new in vitro model applied ^90^Y microspheres to study the effects of low dose beta radiation on colorectal cancer cell line in various oxygenation conditions

**DOI:** 10.1038/s41598-021-84000-7

**Published:** 2021-02-24

**Authors:** Piotr Piasecki, Aleksandra Majewska, Jerzy Narloch, Maciej Maciak, Klaudia Brodaczewska, Michal Kuc, Halina Was, Marek Wierzbicki, Krzysztof Brzozowski, Piotr Ziecina, Andrzej Mazurek, Miroslaw Dziuk, Edward Iller, Claudine Kieda

**Affiliations:** 1 Department of Interventional Radiology, Military Institute of Medicine, Szaserow 128, 01-141 Warsaw, Poland; 2 Laboratory of Molecular Oncology and Innovative Therapies, Military Institute of Medicine, Warsaw, Poland; 3 Postgraduate School of Molecular Medicine, Medical University of Warsaw, Warsaw, Poland; 4 Radiological Metrology and Biomedical Physics Division, National Centre for Nuclear Research, Otwock, Poland; 5 Department of Nuclear Medicine, Military Institute of Medicine, Warsaw, Poland; 6 POLATOM Radioisotope Centre, National Centre for Nuclear Research, Otwock, Poland

**Keywords:** Cancer microenvironment, Cancer therapy, Tumour heterogeneity

## Abstract

We propose a new in vitro model to assess the impact of ^90^Y-microspheres derived low-dose beta radiation on colorectal cancer cell line under various oxygenation conditions that mimic the tumor environment. Cancer cells (HCT116) proliferation was assessed using Alamar Blue (AB) assay after 48, 72, and 96 h. FLUKA code assessed changes in cancer cell populations relative to the absorbed dose. In normoxia, mitochondrial activity measured by Alamar Blue after 48–72 h was significantly correlated with the number of microspheres (48 h: r = 0.87 and 72 h: r = 0.89, *p* < 0.05) and absorbed dose (48 h: r = 0.87 and 72 h: r = 0.7, *p* < 0.05). In hypoxia, the coefficients were r = 0.43 for both the number of spheres and absorbed dose and r = 0.45, r = 0.47, respectively. Impediment of cancer cell proliferation depended on the absorbed dose. Doses below 70 Gy could reduce colorectal cancer cell proliferation in vitro. Hypoxia induced a higher resistance to radiation than that observed under normoxic conditions. Hypoxia and radiation induced senescence in cultured cells. The new in vitro model is useful for the assessment of ^90^Y radioembolization effects at the micro-scale.

## Introduction

A number of randomized controlled clinical trials have shown a positive effect of selective internal radiation therapy (SIRT) on response to treatment, in terms of time to progression and progression-free survival in metastatic colorectal cancer (mCRC) in the liver. However, overall survival remains unaffected^1–3^.

**Figure 1:**
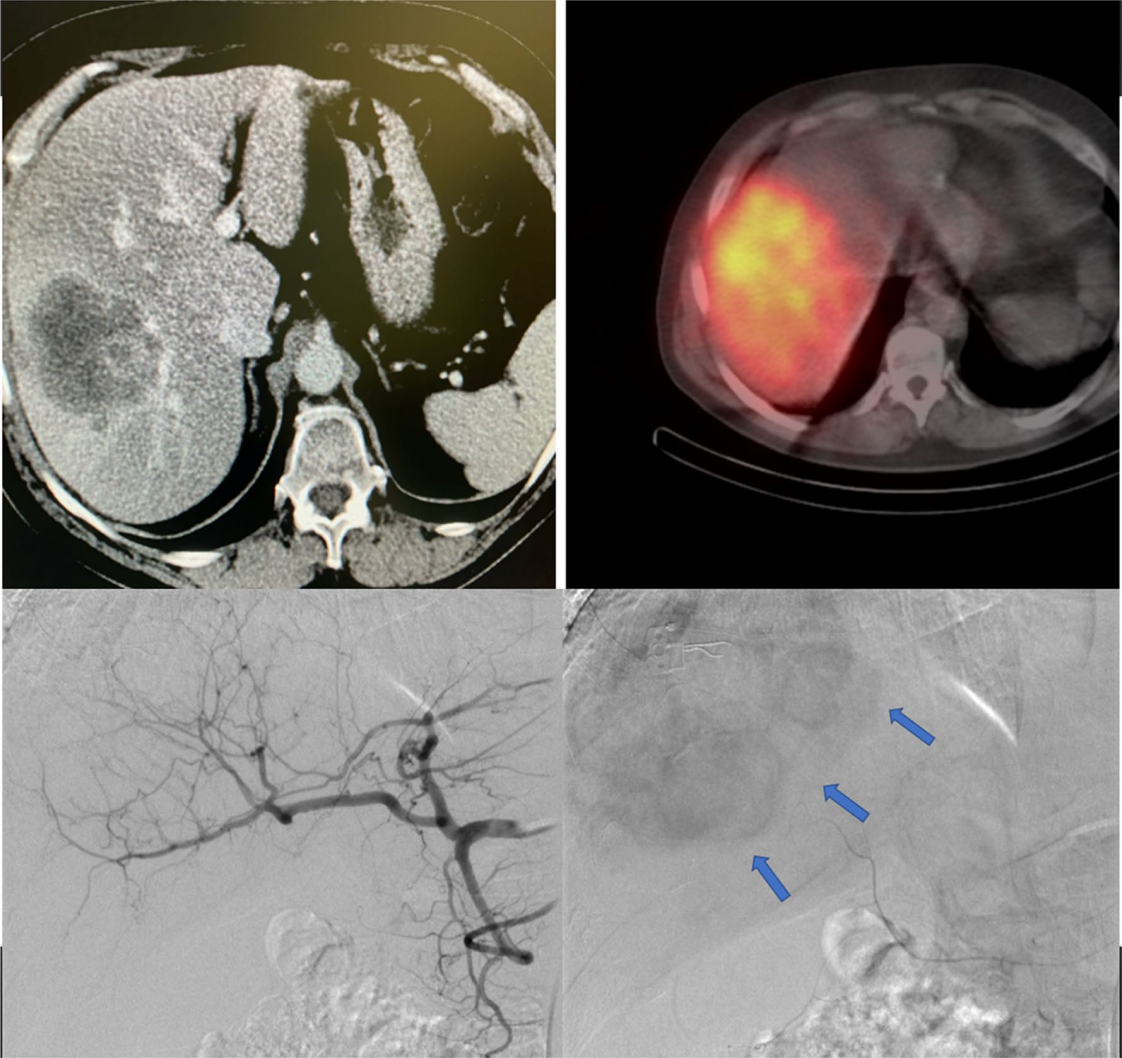
Non-homogenous areas in a solid liver tumor (mCRC) in contrast-enhanced computed tomography and non-homogenous spread of beta-emitting microspheres within a tumor in SPECT/CT (**A**,**B**). Angiogram of hepatic arteries and hepatic tumors contrast enhancement—blue arrows (**C**,**D**). Images of one of the patients treated with radioembolization in our center (with permission).

In SIRT treatment, Yttrium-90 is used as a source of beta radiation, which is bound to resin or glass microspheres injected into the hepatic artery. The ^90^Y-microspheres are preferentially embedded into liver tumors (for example mCRC) as branches of the hepatic artery supply them better than that in healthy liver (ratio 3:1). The spread of ^90^Y-microspheres within the tumor is not homogenous and varies from tumor to tumor according to the quality and density of the vasculature (Fig. 1). The key point of treatment is to calculate ^90^Y activity, which can destroy cancer without harmful effects on healthy liver parenchyma. Improvements in the efficacy of SIRT could be achieved by dose individualization or by a better understanding of the mechanisms responsible for radiation resistance at a micro-scale^4–7^. Yttrium-90 is a pure high-energy beta emitter with a half-life of 64 h; therefore, microspheres can deliver a therapeutic radiation dose for approximately 2 weeks after the injection. It is believed that an absorbed dose higher than 70 Gy would be efficient in destroying colorectal metastatic tumors^8,9^.

Beta radiation can affect cells by direct or, more often, indirect action. Primarily, as soon as a strand of DNA is damaged, cancer cells begin the process of self-destruction known as apoptosis^10–13^. In the indirect action, beta radiation, that is, high-speed electrons lose their energy when passing through cells and transfer this energy to atoms of molecules along its track. This results in the excitation or ionization of atoms. Radiation-induced ionization creates very unstable and reactive free radicals, which can destroy cells by initiating harmful chemical reactions inside the cells^10,12,13^. Although radiotherapy is one of the most efficient therapies utilized in oncology, further investigation is needed to explain the mechanisms of resistance to radiation within cancer cells. One of these mechanisms is thought to be DNA damage repair or repopulation^13^. A key contributor to tumor resistance to radiation may be the oxygenation level at which the tumor grows. This means that in lower oxygen conditions, many tumor cells are able to survive by developing mechanisms of adaptation to both hypoxia and radiation^14–16^. Indeed, toxicity relies on free radical formation, which depends on the availability of oxygen since these are mainly oxygen species. These processes participate in the selection of hypoxia-tolerant and hypoxia-intolerant apoptosis-resistant cancer cells^17^. This feature may be transmitted between cancer cells by extracellular vesicles^17^ and may finally lead to the clinical failure of radioembolization.

To date, data on the effects of radiation on cancer cells are derived mostly from models that use an external beam radiation source. To the best of our knowledge, there is a lack of in vitro experiments utilizing beta-emitting ^90^Y-microspheres on human cancer cell lines. Naturally inhomogeneous dispersion after intra-arterial injection of ^90^Ymicrospheres in the liver can be successfully reflected in placing them on cancer cell culture plates^18^ where they randomly spread among the cells. The cells are differently affected by the radiating microspheres as a function of their relative distance. Similarly, in vivo, ^90^Y microspheres located directly among colorectal cancer cells can irradiate them continuously as this takes place after the radioembolization procedure^7^. Constant beta radiation may affect cancer cells differently than one-dose (or even multi-dose) exposure to an external beam. Especially, when the defense mechanisms used by cancer cells to survive need time to develop (i.e., repopulation or DNA strand repair)^13^. Data from in vitro-based research on the effects of beta radiation on colorectal cancer cell lines could lead to the improvement of radioembolization results in mCRC patients in whom impressive elongation of time to disease progression is obtained but without a change in overall survival time^2^.

Taking into account the strong potential effect of the level and the reaction of oxygen on the mechanism of beta radiation action in a cell, this study aimed to prove it directly. This was done by assessing the effect of low beta radiation on the growth of colorectal cancer cells under different oxygen conditions in vitro*,* reflecting physiologic oxygen tension and pathologic hypoxia, as compared to the classically used non-physiological, normoxic conditions.

## Methods

### Conception of a novel in vitro model for SIRT

**Figure 2:**
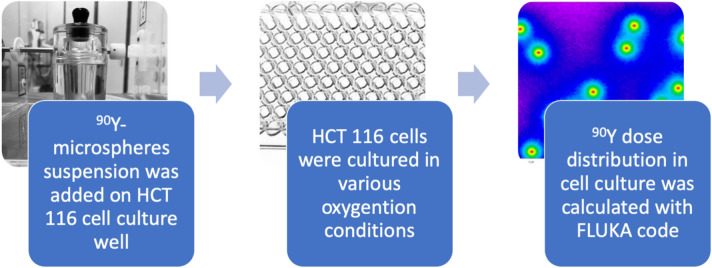
Conception of a novel in vitro model for SIRT. ^90^Y microspheres were delivered in dedicated lead-glass vials (left image), transferred to HCT116 cell plates, which were cultured in various oxygenation conditions. FLUKA simulation software was used to calculate dose distribution across the plate well (right image).

To assess the effects of ^90^Y-radioembolisation in vitro, a new model utilizing HCT116 cell line human colon carcinoma was developed^19^. The suspension of resin ^90^Y-microspheres was added to 96-well, HCT116 cell culture plates. In this way, a random non-homogenous spread of beta-emitting microspheres within colorectal cancer was mimicked, with continuous irradiation of the cancer cells, as in SIRT treatment. Assessment of cancer cell proliferation and other microscopic observations were done after 48, 72, and 96 h. In order to link changes in the cancer cell population and morphology with absorbed dose after beta radiation, the FLUKA code was used for the first time in in vitro calculations (Fig. 2)—refer to the “Absorbed Dose Calculation” section.

### Microspheres

SIR-Spheres is a medical device consisting of biocompatible microspheres containing yttrium-90, with sizes ranging from 20 to 60 microns in diameter. Yttrium-90 is a high-energy pure beta-radiation emitting isotope with no primary gamma emission. The maximum energy of the beta particles is 2.27 meV with a mean of 0.93 meV. The maximum range of these emissions in the tissue is 11 mm with a mean range of 2.5 mm. The half-life of yttrium-90 is 64.1 h. In use, for the isotope to decay to infinity, 94% of the radiation is delivered in 11 days, leaving only background-level radiation. Manufacturer: Sirtex Medical Limited, 16 Mars Road, Lane Cove, NSW, 2066, Australia^7,20^.

### Cell line

Human colon HCT116 cancer cells were cultured in McCoy's medium (Lonza, Basel, Switzerland) supplemented with 10% fetal bovine serum (Biovest, Tamba, USA), 100 units/mL of penicillin, 100 μg/mL of streptomycin, and 25 μg/mL of amphotericin B (Antibiotic-Antimycotic, Thermo Fisher Scientific, Waltham, Massachusetts, USA). Cells were passaged at 80% confluence by detaching with Trypsin (0.25%) EDTA solution (VWR International, Radnor, Pennsylvania, USA). Cells were Mycoplasma free, as assayed with PCR Mycoplasma Test (PromoCell, Heidelberg, Germany).

HCT116 cells were kindly provided by Dr. Bert Vogelstein (Johns Hopkins University, Baltimore, MD, USA). The batch of cells used in our laboratory was used for the experiments published before^21–23^. In January 2021, HCT116 cell line was authenticated using Short Tandem Repeat (STR) analysis as described in 2012 in ANSI Standard (ASN-0002) Authentication of Human Cell Lines; the submitted profile was reported similar to the following ATCC human cell line(s): CCL-247.

### Cell culture

**Figure 3:**
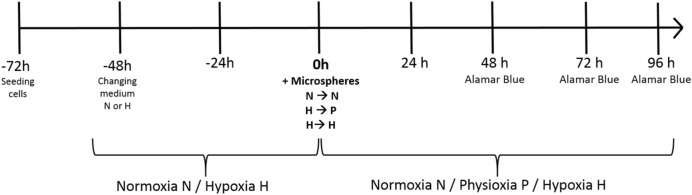
Experiment protocol. (N- Normoxia 19% O_2_, P- Physioxia 5% O_2_, H- Hypoxia 1% O_2_).

3400 cells/cm^2^ were seeded on 96-well cell culture plates and allowed to adhere to the culture surface under standard normoxic conditions (N) (37 °C, 19% O_2_, 5% CO_2_). At the same time, the culture medium (McCoy’s with 10% FBS) was placed in normoxic and hypoxic incubators to obtain a pre-balanced, properly oxygenated medium for further experiments. After 24 h, the medium in culture was changed to pre-balanced normoxia or hypoxia, and the cells were placed in normoxic or hypoxic (H) conditions in the Xvivo X3 workstation (Biospherix, Parish, New York, USA) (37 °C, 1% O_2_, 5% CO_2_) for another 48 h. Thereafter, the medium was changed again to a normoxia, hypoxia, or physioxia (P) pre-balanced medium, and a 10% volume of radiating microspheres was added (^90^Y-microspheres, SIR-Spheres, Sirtex, Australia). Cells were cultured for 48, 72, or 96 h with ^90^Y-microspheres, pretreated in normoxia under normoxic conditions, while those pretreated in hypoxia under physioxic (37 °C, 5% O_2_, 5% CO_2_) or hypoxic conditions. To minimize the impact of beta radiation on HCT116 cells, plates were cultured separately with the space between them reaching at least 10 cm. Dosimetry of the plates and laboratory space was also evaluated. The experimental protocol is represented as a scheme (Fig. 3).

### Alamar Blue assay

To measure cell growth based on mitochondrial activity, the Alamar Blue assay (G-Bioscience, USA) was performed after 48, 72, and 96 h of culture with radiating microspheres according to the manufacturer's instructions. Absorbance was measured after 3 h using a plate spectrophotometer (MultiscanGO, ThermoFisher Scientific, Waltham, Massachusetts, USA) at 570 and 600 nm. The percentage of reduced Alamar Blue was calculated compared to cells without microspheres, and it was shown as a percentage relative to the control due to the differences in metabolic activity between oxygen conditions in control cells, without microspheres (data not shown).

Additional assays (BrdU and BGal) were used when initial observations of senescent cells were performed.

### Cell proliferation ELISA BrdU Assay

BrdU incorporation assay (Sigma-Aldrich, Saint Louis, Missouri, USA) was used to assess cell proliferation based on DNA synthesis 96 h after exposure to radiating microspheres in normoxia according to the manufacturer’s protocol. Absorbance was measured using a plate spectrophotometer (MultscanGO, ThermoFisher Scientific, Waltham, MA, USA) at 450 nm. Results were compared to those of control cells normalized to 100%.

### Detection of β-galactosidase

SA-β-Gal activity was detected after 96 h in control cells and cells treated with microspheres under normoxic conditions according to the method of Dimri et al^24^. Cells were fixed with 2% formaldehyde and 0.2% glutaraldehyde in PBS, washed, and exposed overnight at 37 °C to a solution containing 1 mg/mL 5-bromo-4-chloro-3-indolyl-b-D-galactopyranoside, 5 mM potassium ferrocyanide, 150 mM NaCl, 2 mM MgCl2, and 0.1 M phosphate buffer, pH 6.0.

### Microscopic observations

Cells were visualized using an inverted microscope; Olympus CKX41 with camera UC30 and Olympus Entry Cell Sense 1.8.1. software (Olympus, Tokyo, Japan). The approximate number of microspheres per well was calculated from the photos.

All experiments were repeated two times.

### Absorbed dose calculation

**Figure 4:**
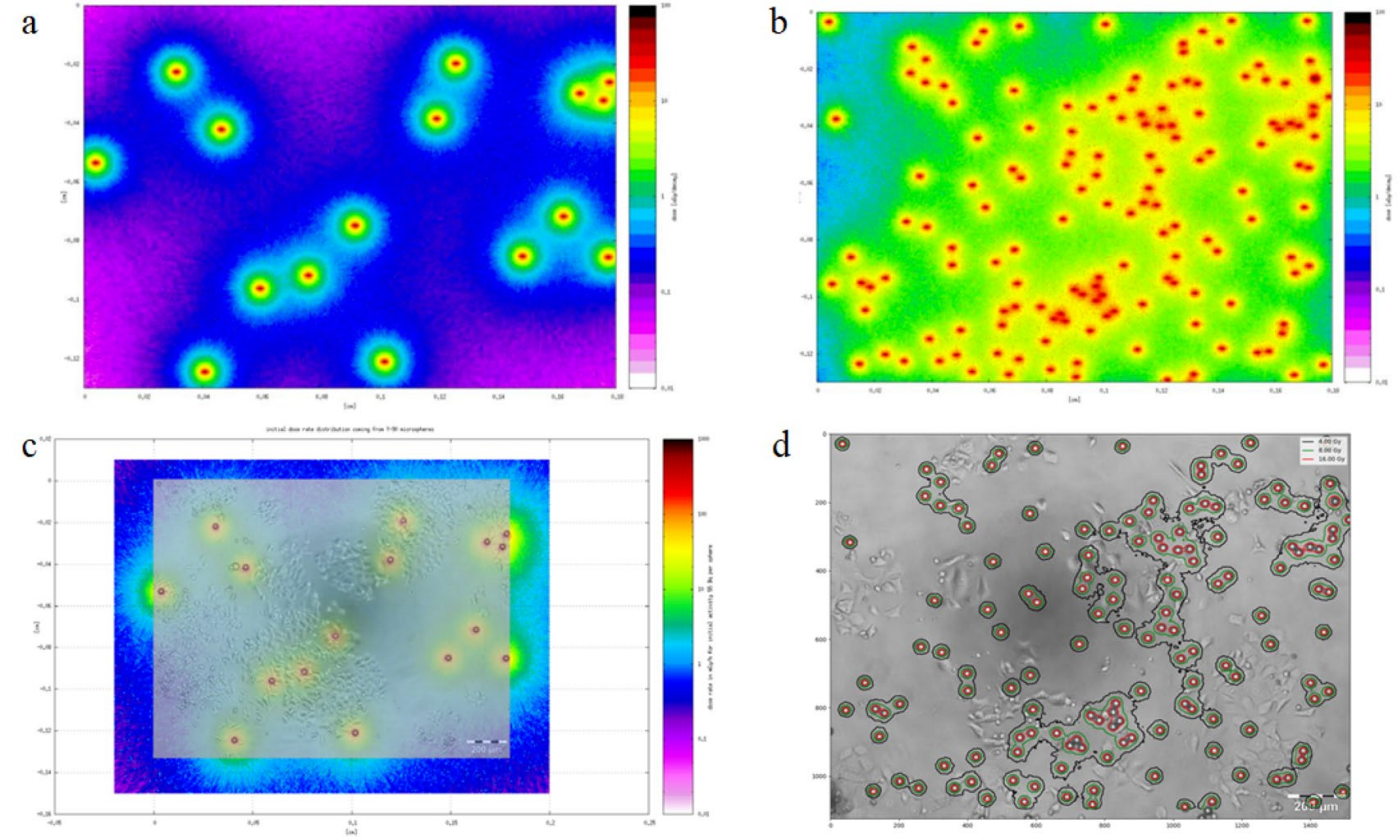
Absorbed dose distribution in cell culture wells shown with the FLUKA simulation package. (**a**,**b**) showing total absorbed dose distribution for a variable number of spheres. (**c**) shows superposition of the microscopic image of cells and microspheres, and simulated dose rate distribution coming from the spheres. (**d**) illustrates dose distribution on the microscopic image as isodoses. Software: FLUKA2011 Version 2x.4 Nov-18 (https://www.fluka.org/fluka.php?id=secured_intro).

To calculate the dose distribution in the cell cultures with a varying number of ^90^Y-microspheres administered in the sample, the FLUKA code was used (Fig. 4)^25,26^. The FLUKA code is a Monte Carlo tool for calculations of particle transport and interactions with matter, which can be used for medical applications^27–30^.

From all the samples that were analyzed in the study, three representative cases with a distinct number of ^90^Y- microspheres were chosen: 15 ± 1, 149 ± 1, 216 ± 1. The microscope field of view (FOV), 1800 µm × 1300 µm, was the same in all cases. Based on the microscopic images of the samples; the geometric models reflecting the distribution of ^90^Y-microspheres in the samples were defined. For the numerical model, the following assumptions were made: the medium in which the ^90^Ymicrospheres were placed was defined as filled with pure water, the thickness of which was equal to the microsphere diameter. ^90^Y-microspheres were defined as spheres with a diameter of 32.5 µm (median diameter of the Sirtex spheres) and specific activity *A*_*0*_ of 55 Bq per sphere. Each source of radiation was defined as homogeneously distributed inside the sphere, not only on the surface of the sphere. As an estimator for the absorbed dose calculation, the cuboid of dimensions equal to the FOV times sphere diameter was defined. Since the sampling algorithm was implemented in the calculation code, for each specific sample, the normalization factor equal to the number of spheres in the FOV was applied. The results of the simulation were expressed as absorbed dose *D*_*calc*_, normalized to one radioactive decay. Additionally, in order to calculate the accumulated absorbed dose *D*_*t*_ obtained after a certain period of time *t*, final calculations according to Eq. (1) were necessary:

1 $$ D_{t} = D_{calc} \left[ {\frac{{A_{0} }}{\lambda }\left( {1 - e^{ - \lambda t} } \right)} \right], $$

Based on the *D*_*calc*_ obtained for the described cases above, and considering that the standard deviation of the mean was at the level of 1.4%, for the remaining samples in the study, one common factor *D*_*calc*_ was calculated and applied for final calculations based on Eq. (1). This was defined as the average value dose.

### Definitions

*Normoxia* the “normal” oxygen levels in tissue culture (18.75% at the level of Warsaw oxygen in H_2_O saturated atmosphere and at 37 °C).

*Physioxia* “physiologic oxygen tension” in tissues, ranging from 13% in the lungs to 1% in the dermo-epidermal junction^31^.

*Hypoxia* level of oxygen tension lower than that in physioxia. The level of the partial pressure of oxygen here is 1%, corresponding to the values found in the growing tumor site^32^.

## Results

### New in vitro model

There was no beta radiation contamination. Beta radiation from cultured plates was similar to background radiation. A suspension of beta-radiating microspheres on HCT116 cell culture 96-well plates was feasible, safe, and efficient. Cultivation of cells in various oxygen conditions partly reflected the heterogeneity of the tumor and allowed for assessment of the potential response of cells located in different tumor zones (with different vasculature).

### Effect of radiation on cell growth

Significant differences were observed between the growth of the HCT116 cell line in the control compared to that in cells treated with ^90^Y-microspheres in normoxia, hypoxia, and hypoxia-physioxia after 48, 72, and 96 h. Tables 1 and 2.

**Table 1:** Descriptive statistics for observations performed in various oxygenation conditions.

Conditions	Time	Variable	Mean	SD	*p*-value
Normoxia	48 h	Reduced AB (% control)	66.3	6.9	0.0009
		No. of spheres	69	109	–
		absorbed dose (Gy)	14.4	22.8	–
	72 h	Reduced AB (% control)	70.4	11.5	0.004
		no. of spheres	92	124	–
		absorbed dose ()	20.8	25.5	–
	96 h	Reduced AB (% control)	60.8	14.8	0.067
		No. of spheres	87	11	–
		absorbed dose (Gy)	29	3.5	–
Hypoxia	48 h	Reduced AB (% control)	66.9	3.9	0.001
		no. of spheres	59	62	–
		absorbed dose (Gy)	12.4	13.1	–
	72 h	Reduced AB (% control)	60.2	11.1	0.001
		no. of spheres	93	153	–
		absorbed dose (Gy)	22.5	32.8	–
	96 h	Reduced AB (% control)	46.9	6.5	0.067
		No. of spheres	122	42	–
		absorbed dose (Gy)	41	14.3	–
Hypoxia—physioxia	48 h	Reduced AB (% control)	70.9	5.1	0.0002
		no. of spheres	53	52	–
		absorbed dose (Gy)	11.1	11.2	–
	72 h	Reduced AB (% control)	67.2	5.1	0.03
		No. of spheres	59	46	–
		absorbed dose (Gy)	16.6	12.9	–
	96 h	Reduced AB (% control)	56.4	5.4	0.067
		No. of spheres	165	21	–
		absorbed dose (Gy)	55	9.7	–

*AB* Alamar Blue, *SD* standard deviation. Significance of reduced AB (% control) compared to that in relative control in subsequent time frames in different oxygen conditions.

^1^Wilcoxon signed-rank test.

**Table 2:** Comparisons of mean of reduced Alamar Blue in different oxygen conditions at different time frames.

Time frame	Conditions	*p*-value^1^
48 h	N vs. H	0.03
	N vs. H-P	0.21
72 h	N vs. H	0.07
	N vs. H-P	0.005
96 h	N vs. H	0.89
	N vs. H-P	0.89

*N* normoxia, *H* hypoxia, *H-P* hypoxia-physioxia. For details, see the “Methods” section.

^1^Wilcoxon signed-rank test.

**Figure 5:**
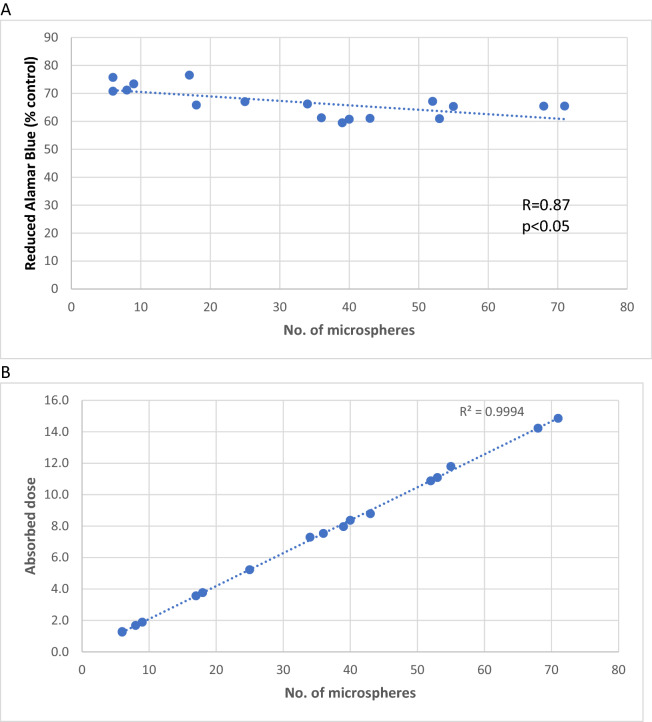
A. Correlation between number of microspheres and mitochondrial activity measured by Alamar Blue in normoxia after a 48-h exposure. B. Correlation between number of microspheres and absorbed dose in normoxia after a 48-h exposure.

In normoxia, the mitochondrial activity measured by Alamar Blue after 48 h was significantly correlated with the number of spheres (r = 0.87, *p* < 0.05) (Fig. 5A) and absorbed dose (r = 0.87, *p* < 0.05) (Fig. 5B). Observations were analogous for a 72 h incubation (r = 0.89 and r = 0.87, respectively; *p* < 0.05). In hypoxia, the coefficients were weaker compared to those in normoxia when observations were done after 48 and 72 h, yet remained significant (r = 0.43, *p* < 0.05) for both the number of spheres and absorbed dose (r = 0.45, r = 0.47, respectively, *p* < 0.05).

Observations made in mixed conditions—hypoxia-physioxia—closely resembled those made in normoxia, and showed a stronger correlation after a 48-h incubation (r = 0.9 and r = 0.91, for number of spheres and absorbed dose, respectively; *p* < 0.05). Coefficients for 72-h incubation maintained a tendency towards r = 0.86 and r = 0.8, respectively (*p* < 0.05). Coefficients were not statistically significant after 96 h for each oxygen condition.

**Figure 6:**
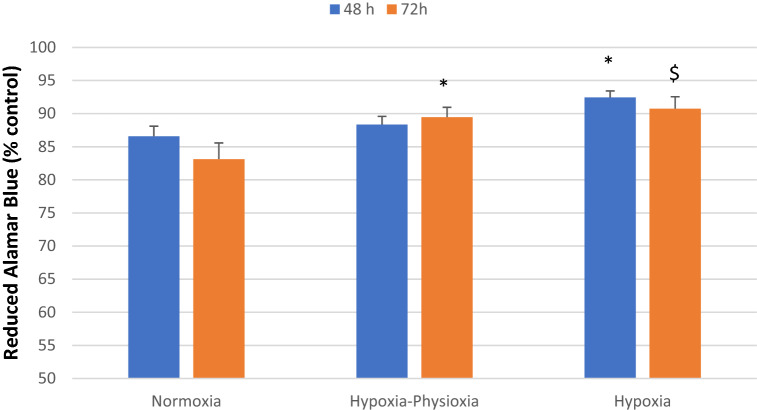
Effect of distinct oxygen tension conditions on cell sensitivity to radiation in vitro at different time points. Cell viability, measured by Alamar Blue Assay relative to the control (of untreated cells in corresponding oxygen condition), as a percentage is shown after 48 and 72 h of exposure to beta radiation. N = 2 biological replicates in 6 technical replicates; bars show mean + /- SEM, *p value < 0.05, ^$^*p* value < 0.1 in comparison to normoxia.

The mitochondrial activity measured after 48 h was significantly lower in hypoxia when compared to high that in oxygen tension (*p* = 0.03). These differences were not statistically significant when compared to those in mixed conditions (*p* = 0.21). After 72 h, these values were significant (*p* = 0.07 and 0.005, respectively). Detailed comparisons are shown in Table 3 and Fig. 6. For wells in which the absorbed dose exceeded 70 Gy, the mean reduced Alamar Blue value was 53% for normoxia after 48 h, 13% for normoxia after 72 h, and 13% for hypoxia after 72 h. The percentage of wells in which the absorbed dose was higher or lower than 10 Gy or 70 Gy are presented in Table 3.

**Table 3:** Percentage of wells in which absorbed dose (AD) was higher or lower than 10 Gy or 70 Gy.

Time frame	Conditions	AD mean (Gy)	AD > 70 Gy (%)	10 Gy < AD < 70 Gy (%)	AD < 10 Gy (%)
48 h	N	14	3	31	66
	H-P	11	0	45	55
	H	12	0	46	54
72 h	N	21	9	48	43
	H-P	16	0	50	50
	H	22	3	50	47
96 h	N	29	0	100	0
	H-P	55	12	88	0
	H	41	0	100	0

Differences in the parentage of reduced Alamar Blue for wells in which the absorbed dose was higher or lower than 10 Gy are presented in Table 4.

**Table 4:** Differences in the percentage of reduced Alamar Blue in wells in which absorbed dose (AD) was higher or lower than 10 Gy. Calculations for 96 h were not done.

Time frame	Conditions	10 Gy > AD Median (%)	10 Gy < AD Median (%)	P U Mann
48 h	N	60.5	70.9	0.001
	H-P	65.1	75.1	> 0.001
	H	68.6	66.7	ns
72 h	N	60.4	80.1	> 0.001
	H-P	62.1	72.3	> 0.001
	H	59,9	62,5	ns

### Effect of radiation on cell morphology

No change in the typical morphology of HCT116 cells was observed after 48, 72, and 96 h of growth in normoxia.

**Figure 7:**
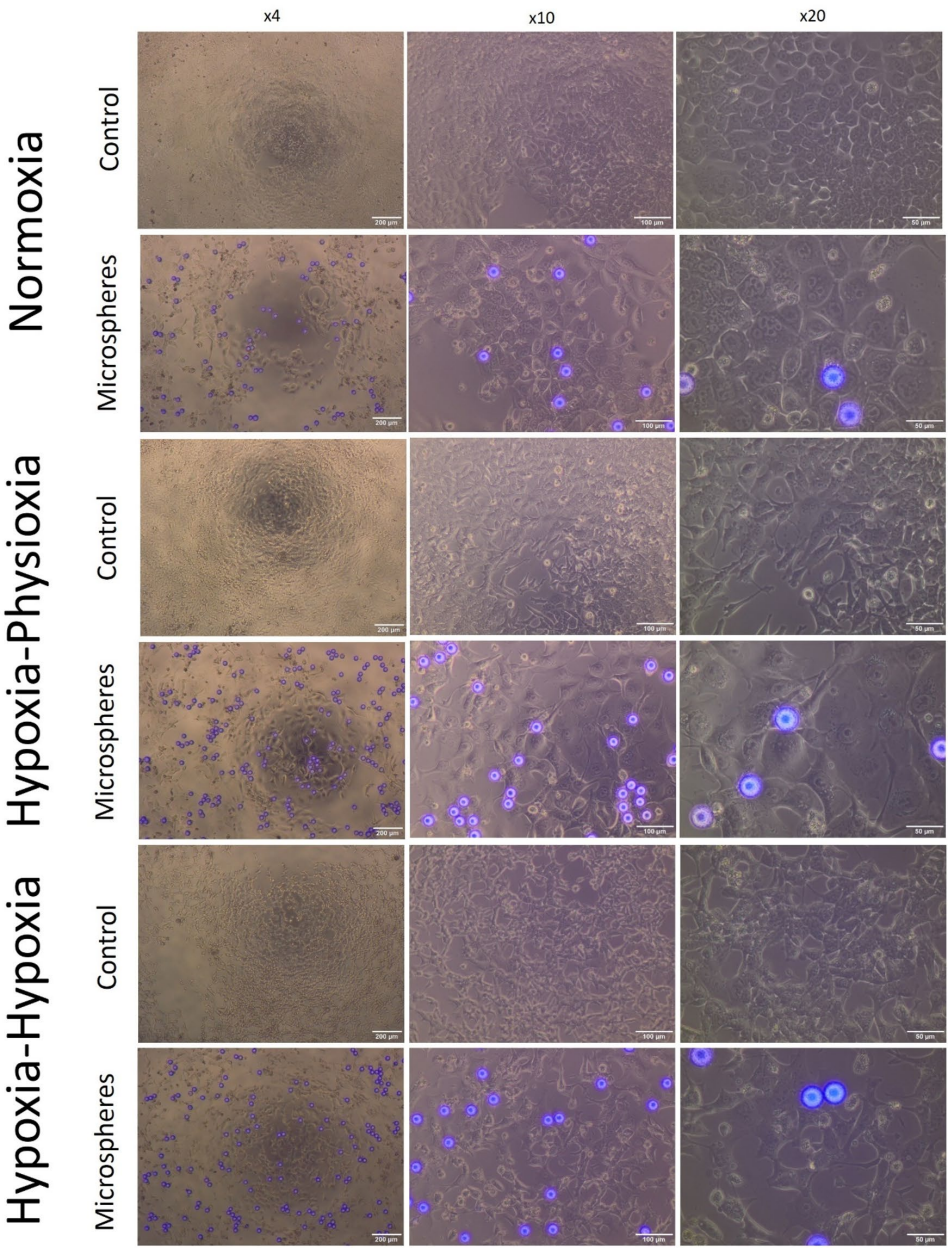
Microscopic observations of control cells treated with microspheres (after 96 h) under several magnifications (× 4, × 10, × 20) in various oxygenation conditions (compared to the corresponding control). Fluorescent spheres are ^90^Y-microspheres.

**Figure 8:**
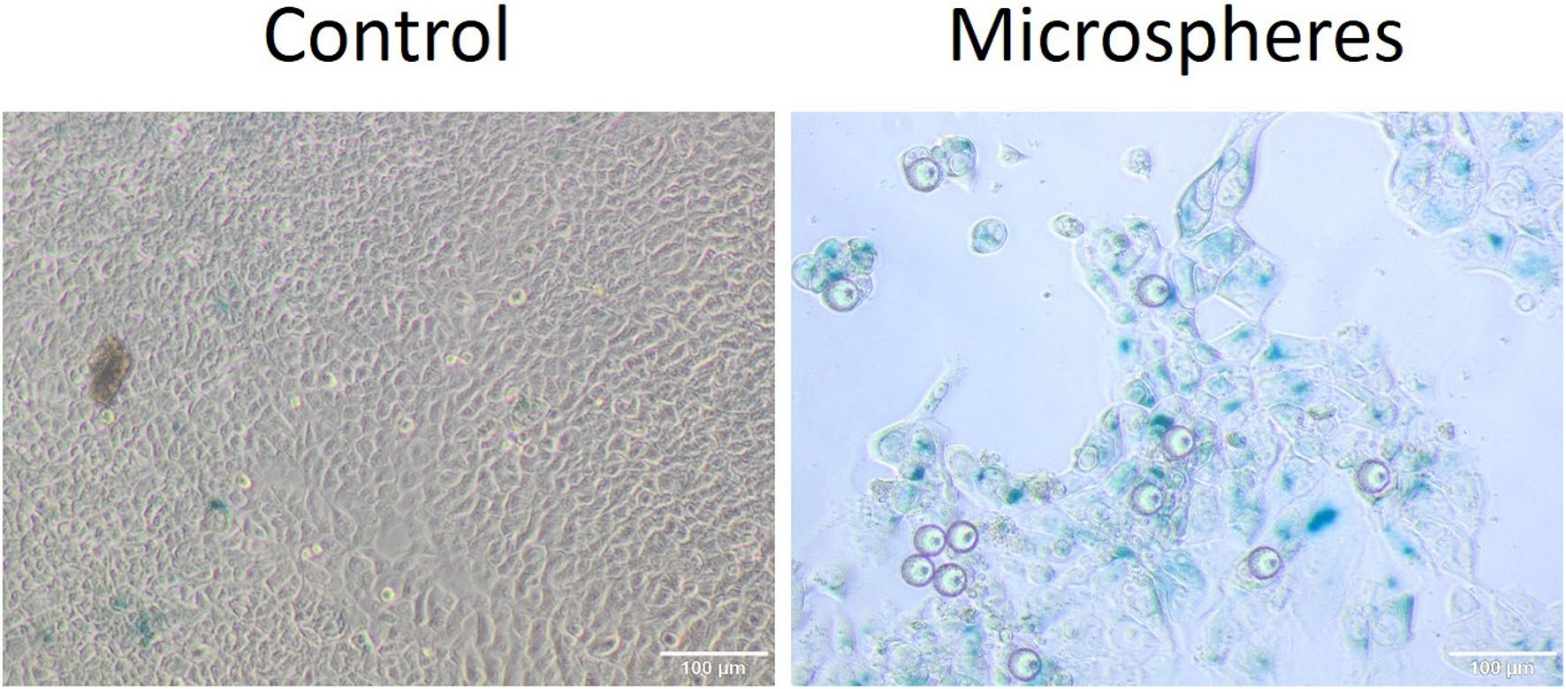
Detection of senescent cells using SA-β-gal staining in normoxia after 96 h (right: control, left senescent cells in culture growth with 90Y microspheres).

In cultures where the cells were in the presence of radiating microspheres, growth arrest, size increase, and nucleus granularity, as well as polyploidization were observed. A supplementary video of culture growth is provided. Hypoxia yielded a number of larger cells displaying polyploidic nucleoli, a feature observed in cancer cell senescence (Fig. 7). A number of such cells have been observed in hypoxia and hypoxia-physioxia. The occurrence of senescent cells was confirmed by SA-β-gal staining after treatment in normoxia (Fig. 8).

## Discussion

To the best of our knowledge, there are no in vitro models to investigate the effects of beta radiation on colorectal cancer cells. Data derived from studies on external beam radiation therapy would not fully explain the processes occurring during ^90^Y radioembolization, which could be regarded as brachytherapy utilizing beta radiation. ^90^Y radioembolization has been widely used without extensive research on the impact of beta radiation on human liver and cancer cells. The first substantial report regarding this issue was done by American researchers on patients who underwent liver transplantation due to mCRC or HCC tumors^18^. The effect of liver tumor irradiation was assessed depending on the amount of radiated particles gathered non-homogenously in the solid tumor. As shown by isodose curves within the tumor, the higher the absorbed dose, the greater the amount of tumor necrosis observed. These results could translate into the destruction of cancer cells, necessitating very high absorbed radiation doses^18^. Areas with insufficient numbers of gathered radiating microspheres occurred, and cancer cells survived the resulting low radiation^18^.

The goal of our study was to evaluate the impact of microspheres emitting low doses of beta radiation on the growth of colorectal cancer cells under variable oxygen tension mimicking the microenvironment of the tumor mass.

We designed a new experimental model utilizing lower absorbed doses sourced from ^90^Y microspheres applied directly to cancer cells. In this way, we tried to create an environment similar to real ^90^Y treatment in which the random spread of radiating microspheres within tumors (consisting of areas of different tissue oxygenation levels) is combined with observations of beta radiation effects on cancer cells applied continuously for several days^33^.

### Absorbed dose and number of radiating microspheres.

Significant differences between the growth of HCT116 cancer cell lines in the control compared to plates with ^90^Y-microspheres under various oxygen conditions were observed in our study. The effects were linked to the number of ^90^Y microspheres and the absorbed dose.

Non-homogenous ^90^Y-microsphere spread within metastatic liver tumors lead to variable concentrations of ^90^Y-particles in different tumor sections, which affected the amount of tumor absorbed dose^18,33^. American researchers reported that colorectal metastases had almost 90% necrosis if the absorbed dose ranged from 100 to 1000 Gy for most of the tumor volume; that is, 10% of tumor cells survived despite high-dose treatment^18^.

We have attempted to observe the outcome of cancer cells that were likely to survive radiation; that is, those that absorbed lower-than-lethal—70 Gy—absorbed dose. After 48 h of cancer cell growth, the average absorbed dose was 14 Gy in normoxia, 11 Gy in hypoxia-physioxia, and 12 Gy in hypoxia (ranging from 0.6 Gy to 113 Gy, *p* > 0.05). In normoxia, only 3% of cell culture wells exceeded 70 Gy, 31% of wells with absorbed doses between 10–70 Gy, and 66% of wells with absorbed doses below 10 Gy. Similar results were observed for hypoxia-physioxia and hypoxia. Despite the suboptimal absorbed doses, these results showed a decline in cancer cell proliferation in all oxygen conditions as compared to those in the control after 48, 72, and 96 h. This means that low beta radiation was the cause of cancer cell death, the effect was more visible with a higher number of ^90^Y-microspheres and a higher absorbed dose. However, resistant cancer cells were observed even in plates with absorbed doses higher than 70 Gy. We could not observe the full spectrum of radiation effects due to the time limitation of cell culture observation (exposure time of 3 days) imposed on us for practical reasons; that is, no need for cell re-passage with radiation contamination. At a given radiation dose, the time of radiation exposure would reach approximately 14 days. It is worth mentioning that both the low and high absorbed doses observed in our study would not be sufficient to completely destroy colorectal tumors in real treatment.

### Hypoxia

Most studies have been conducted on cancer cells cultured in normoxia, which does not reflect the oxygen conditions of the inhomogeneous tumor microenvironment^31^. Thus, our research focused on investigating various oxygenation conditions to mimic those present in tumors. We noticed some differences in cancer cell proliferation depending on the level of oxygen during culture cell incubation. In normoxia and mixed conditions, there were significant differences in cell proliferation between the lower and higher absorbed doses. Contrasting observations were made in hypoxia, in which cells were less susceptible to radiation. In particular, mitochondrial activity was significantly lower after 72 h. This suggests that hypoxia induces protective mechanisms against cell damage caused by beta radiation. A possible explanation is that lower oxygen pressure in the cell microenvironment means a lower probability of free radical creation^15^. In this situation, DNA strand damage remains the main or even the only mode of cell destruction after radiation.

Hypoxia is common in most solid tumors, in which, the amount of oxygen delivered by abnormal vasculature is not adequate to meet the needs of rapidly growing cancer cells^34^. Median liver tumor oxygenation is 0.8% compared to 3.8% in normal liver tissue^32,35^. Tumor hypoxia is an important factor affecting different kinds of therapies, including radiotherapy, which is based on the creation of reactive oxygen species^14,15,34^. Perfusion-limited oxygen delivery in tumors is caused by abnormal vasculature with a lack of the proper layers within arteries and veins, and with irregular architecture. This leads to functional insufficiency of the vessels, resulting in tumor ischemia^15^. Cancer cells develop a number of mechanisms of adaptation to low oxygen levels based on the activation of hypoxia-inducible factors (HIFs). HIFs allow cancer cells to shift to anaerobic energy production by regulating the expression of multiple genes associated with angiogenesis, pH balance, and cell apoptosis or senescence^15^. A three-fold higher dose of radiation is required to destroy hypoxic cancer cells compared to that in normoxic ones^14,15^. An adequate oxygen pressure level is needed precisely at the time of irradiation for effective free radical production in cancer cells (about two weeks in the case of ^90^Y radioembolization). Even a minimal increase in oxygen tension at that time results in an improved radiation effect on cancer cells, as seen in the proliferation results of cells growing in hypoxia-physioxia conditions^15^. The protective effect of hypoxia was clearly reflected in our study. In normoxia, mitochondrial activity measured by AB after 48 and 72 h was strongly correlated with the number of spheres and absorbed dose. In hypoxia, the coefficients were much weaker (r = 0.87 vs. r = 0.43). Slight enrichment of oxygen supply (from 1 to 5%) was sufficient to reach a strong correlation between reduced AB and absorbed dose.

This observation suggests avoiding hypoxia during endovascular liver cancer embolization to increase the effectiveness of local therapy. This is because blood-flow stasis in the liver artery branches during this procedure may lead to hypoxia in tumors and protect cancer cells from death, given the results presented above. In contrast to liver tumor chemoembolization (TACE), radioembolization does not lead to blood flow stasis during the procedure^7,9,36^. This approach was designed to avoid extrahepatic leaks of radiated microspheres in case of blood-flow stoppage in the liver artery. Another concern is that the calculated amount of ^90^Y-microspheres may not be injected in case of blood stasis, jeopardizing sufficient cumulative absorbed dose within the liver tumors^9^.

### Cell senescence

We observed that the cells exposed to radiation showed typical signs of senescence. These were especially conspicuous when cell cultures were exposed to lower oxygen levels. Cells cultured in hypoxia were larger and polyploidy was more prevalent than in those grown in more oxygen-rich conditions.

Cell senescence is a state of arrest of normal cell growth at the end of their life (replicative senescence). Physiologically, it should be a mechanism for the prevention of carcinogenesis. In some conditions, it is possible to cause cells to enter premature senescence induced by various stress factors: i.e. oxygen stress, DNA damage, hypoxia, chemo-, or radiotherapy^16,37,38^. If it occurs during chemotherapy, it may lead to cancer cell resistance and tumor regrowth^21,22^. Senescent cells have some specific features such as growth arrest and resistance to apoptosis, but they remain metabolically active, increase in size and granularity, and have higher senescence-associated beta-galactosidase activity^38^. Typically, senescence-associated secretory phenotype (SASP) consists of various growth factors, cytokines, and enzymes (i.e.IL-1, IL-6, IL-8, and metalloproteinase-3). These factors may have an impact on inflammation, tumor growth, and the appearance of metastases^22,39^. Cancer cell senescence can also be induced by chemotherapy or radiation. Even low-dose gamma-ray exposure may lead to G1 cell cycle arrest. As we can observe in our study, the mean absorbed dose in particular wells was below 70 Gy, which is considered a lethal dose for colorectal cancer. This may be the reason for the presence of large numbers of senescent cells in cancer cultures with radiating microspheres in every low oxygen conditions. This means that in tumors without sufficient absorbed dose after radioembolisation, cell senescence may be induced. These cells may be precursors of new lines, resistant to further systemic therapy, which was proved for senescent cancer cells created after chemotherapy. This topic needs further investigation in our opinion.

### Alamar Blue and BrDu assays

**Figure 9:**
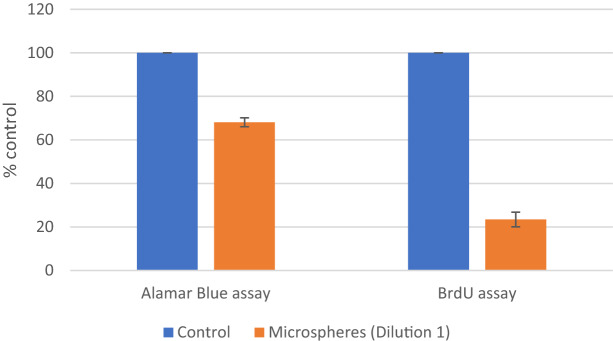
Comparison of different cell viability assays used on the same culture. Effect of microspheres on HCT116 cells proliferation in normoxia after 96 h, differences between Alamar Blue and BrdU. Control was normalized to 100%

Initial assessment of cell viability in all cultures was performed using the Alamar Blue reagent, a test based on the ability of living cells to reduce resazurin to resorufin, which is quantitatively assessed by the absorbance of a fluorescence-based plate reader. Our observations of a considerable prevalence of senescent cells, which were more metabolically active than control cancer cells, rendered the use of Alamar Blue reagent inadequate for the assessment of radiation-exposure cellular changes. Several cultures had to be independently assessed by BrdU proliferation assay, which quantitatively assesses bromodeoxyuridine incorporation into newly synthesized DNA of actively proliferating cells (Fig. 9). Effective change in comparison to that in control was even more pronounced since senescent cells, though metabolically active, do not synthesize new DNA.

## Limitations

Our study is not devoid of limitations. We performed the experiment using only one cell line in 2D culture as an initial study. The tumor microenvironment (TME) is more complicated and complex, including other cellular components (e.g., endothelial cells, immune cells, fibroblasts), 3D shape, and hypoxia. To partly mimic the pathological tumor microenvironment, we used various oxygenation conditions and observed significant changes in cellular response. However, advanced culture methods, containing other TME components, are necessary to investigate the biological effects of the radiating microspheres in vitro. Late characterization of visible changes observed after exposure to radiation rendered the use of different cell viability assays (BrdU) possible only in several cultures. Nevertheless, Alamar Blue-based observations were significant enough to draw relevant conclusions on the effect of radiation on mCRC cells. Next, the physical nature of radiated microspheres rendered it impossible to distribute the microspheres between the wells uniformly. However, this in part reflects the in vivo conditions when radioembolization is performed. Last, the presence of radiation materials in culture cells limited the number of additional investigation pathways, which would normally follow (I. A. flow cytometry). For the same reason, the possibility of cell culture passages and the time of culture growth were limited.

## Conclusions

Absorbed doses below 70 Gy can reduce colorectal cancer cell proliferation in vitro. The degree of reduction of cancer cell proliferation depends on the number of ^90^Y-microspheres and the absorbed dose of radiation. Absorbed doses exceeding 70 Gy did not lead to complete cessation of cell proliferation within 96 h of observation. Hypoxia induced a higher resistance to radiation than that observed under normoxic conditions. Cell viability after radiation exposure would presumably be better assessed by new DNA-based assays. Both hypoxia and radiation induced senescent cells in the culture.

## Data Availability

The datasets generated during and/or analysed during the current study are available from the corresponding author on reasonable request.
